# Challenges in Modeling Human Neural Circuit Formation via Brain Organoid Technology

**DOI:** 10.3389/fncel.2020.607399

**Published:** 2020-12-03

**Authors:** Takeshi K. Matsui, Yuichiro Tsuru, Ken-ichiro Kuwako

**Affiliations:** Department of Neural and Muscular Physiology, Shimane University School of Medicine, Izumo, Japan

**Keywords:** human brain organoid, circuit formation, neural differentiation, neuronal migration, axonal projection, synapse formation

## Abstract

Human brain organoids are three-dimensional self-organizing tissues induced from pluripotent cells that recapitulate some aspects of early development and some of the early structure of the human brain *in vitro*. Brain organoids consist of neural lineage cells, such as neural stem/precursor cells, neurons, astrocytes and oligodendrocytes. Additionally, brain organoids contain fluid-filled ventricle-like structures surrounded by a ventricular/subventricular (VZ/SVZ) zone-like layer of neural stem cells (NSCs). These NSCs give rise to neurons, which form multiple outer layers. Since these structures resemble some aspects of structural arrangements in the developing human brain, organoid technology has attracted great interest in the research fields of human brain development and disease modeling. Developmental brain disorders have been intensely studied through the use of human brain organoids. Relatively early steps in human brain development, such as differentiation and migration, have also been studied. However, research on neural circuit formation with brain organoids has just recently began. In this review, we summarize the current challenges in studying neural circuit formation with organoids and discuss future perspectives.

## Introduction

Human brain tissue is considered the best resource for analyzing the mechanisms of human brain development and diseases. However, ethical issues have prevented us from easily accessing human brain tissue samples, especially samples from living patients. Thus, researchers are mainly dependent on human postmortem brains and the brains of aborted fetuses to study the human brain. Additionally, rodent model animals have been used for tissue cultivation and genetic manipulation studies. However, approximately one decade ago, human pluripotent stem cell-derived retinal organoids composed of multiple cell types that imitate the three-dimensional (3D) structure of the retina were reported for the first time (Eiraku et al., 2011). This organoid technology paved the way for analyses of various cell-cell interactions in 3D human tissues *in vitro*. Many protocols for the generation of human organoids that imitate various tissues, such as colon, kidney, lung and liver tissues, have since been published (Jung et al., 2011; Sato et al., 2011; Takebe et al., 2013; Taguchi et al., 2014; Takasato et al., 2014; Dye et al., 2015). In 2013, Lancaster et al. reported the first method for inducing human cerebral organoids that recapitulate the structure of the ventricles and the neuronal layers of the human cerebral cortex, even though these organoids harbor other various brain regions (Lancaster et al., 2013). Following this report, many protocols for generating organoids that mimic other brain components, including the midbrain (Jo et al., 2016), brainstem (Eura et al., 2020), choroid plexus (Pellegrini et al., 2020), cerebellum (Muguruma et al., 2015), and spinal cord (Ogura et al., 2018; Duval et al., 2019), were established. Furthermore, fine-tuned protocols for producing cerebral cortex-specific organoids were later reported (Paşca et al., 2015; Qian et al., 2016, 2018). These brain organoids have been used to study many diseases, such as lissencephaly (Iefremova et al., 2017), Zika virus infection (Qian et al., 2016), and ischemia (Paşca et al., 2019). Additionally, human brain organoids have contributed to investigations of neural development, such as differentiation (Paşca et al., 2015; Qian et al., 2016; Trujillo et al., 2019) and migration (Birey et al., 2017). The formation of precise neural networks and interactions between specific neurons are indispensable for information processing, but limited research on neural circuits has been conducted with human brain organoids. Direct investigation of neural circuits through the use of human organoids will contribute to the elucidation of the mechanisms that underlie the properties of human circuits. This approach will also facilitate the understanding of the mechanisms behind neural circuit-associated developmental diseases.

The developing human brain possesses human-specific cells, structures, gene expression profiles and functions. There is a 1,000-fold difference in brain volume and the number of cells in the brain between humans and mice (Hodge et al., 2019). In humans, outer radial glial cells, which are rare in rodents, produce a large number of cortical layer neurons and enable marked expansion and high complexity of the human cerebral cortex (Hansen et al., 2010; Pollen et al., 2015). *ARHGAP11B*, a human-specific gene that specifically expressed in radial glial cells, may contribute to this expansion in humans (Florio et al., 2015). In addition, human neural circuits are apparently distinct from those of mice. For example, humans harbor unique neuronal circuits around the caudate nucleus and anterior putamen that are associated with executive function and social/language regions in the striatum; however, these circuits are absent in mice (Balsters et al., 2020). In the spinal cord, the pyramidal tract descends from the motor cortex through the lateral column in humans and through the dorsal column in rodents (Lemon, 2008). Some human pyramidal neuron axons make direct connections with spinal motor neurons that are responsible for fine motor skills; however, in mice, pyramidal neuron axons mainly contact motor neurons via interneurons (Lemon, 2008). In humans, approximately 40% of the axons of retinal ganglion cells (RGCs) do not cross the midline at the optic chiasm to innervate the ipsilateral side, whereas in mice, most RGC axons cross the midline, thereby establishing a structural basis for binocular or monocular vision (Petros et al., 2008). In addition, potent human-specific excitatory connections exist between pyramidal neurons and GABAergic inhibitory interneurons, through which a single action potential from a pyramidal cell can induce polysynaptic GABAergic firing of interneurons (Molnár et al., 2008; Szegedi et al., 2016). Finally, human pyramidal neurons are known to have 4 times more docked vesicles at 2 times larger active zones than rat pyramidal neurons (Molnár et al., 2016). Many of these previous studies on human samples were carried out with biopsy/abortion samples, which have many disadvantages, including multiple genetic backgrounds, inconsistencies in patient age, and damage from surgery and diseases.

Fortunately, we can now employ human brain organoids to acquire genetically homogenous samples and introduce genetic manipulations, such as knockout of specific genes by CRISPR/Cas9-mediated genome editing. Although brain organoids still have the issues of variability and artificial metabolic stress, organoid technology provides a novel approach for investigating human neural circuits. In this review, we discuss previous accomplishments and future research directions regarding the use of organoids for studying neural circuits. Additionally, we address the current limitations of organoid technology in studying neural circuits and means for overcoming these limitations.

## Use of Human Brain Organoids for Research on Topics Other Than Neural Circuit Formation

Currently, there are two major protocols for generating brain organoids from pluripotent stem cells. In the first protocol (Lancaster et al., 2013), pluripotent stem cells are differentiated into brain organoids mostly through intrinsic signals; therefore, almost no additional growth factors are required. This protocol yields a random variety of brain components, such as the retina, hippocampus, cerebral cortex and colloid plexus, in each organoid (Lancaster et al., 2013). Neurons in organoids induced by this protocol mature into glutamatergic excitatory neurons and GABAergic inhibitory neurons after 3 months of cultivation (Matsui et al., 2018). Additionally, oligodendrocytes emerge after 6 months of cultivation and form myelin sheath-like structures around axons in the organoids (Matsui et al., 2018). Furthermore, microglial cells, of mesodermal origin, are also present in the long-cultivated organoids (Ormel et al., 2018). While these cerebral organoids resemble the developing human brain, in which intrinsic signals autonomously orchestrate the stepwise differentiation of each component, the low reproducibility and high variability of the induced brain components in the organoids sometimes limit the use of this protocol. The other major protocol for generating brain organoids requires fine tuning of additional growth factors such as BDNF, GDNF and NT-3. This protocol gives rise to more specific components with less variability than the previously mentioned protocol and is able to selectively induce specific brain components, such as the cerebral cortex, brainstem, midbrain, retina, cerebellum, and basal ganglia, in organoids (Paşca et al., 2015; Qian et al., 2016). The brain organoids generated by these two distinct methods have much in common; both types of organoids contain multiple neural cell populations, such as neural stem cells (NSCs), neurons and astrocytes, and exhibit basic neural structures, with NSCs surrounding ventricle-like cavities and neurons on the surface of NSC region forming layered structures similar to those in the developing human brain.

Organoid technology has provided new insights into human brain development and diseases. For example, in the field of development, studies on organoids have revealed that the *retinoblastoma* gene regulates apoptosis of NSCs and neuronal migration in humans (Matsui et al., 2017). Analysis of developing human brain organoids have revealed that the wrinkled structure of the human brain can be explained by two different mechanical forces that act on different regions of organoids, namely, cytoskeletal contraction at the core and nuclear accumulation at the perimeter (Karzbrun et al., 2018). In addition, molecular analysis of human cerebral organoids has shown that suppression of PTEN signaling is a key mechanism underlying proliferation of neural progenitor cells and subsequent folding of the cerebral cortex specifically in the human brain (Li et al., 2017). Evolutionary analysis comparing gene expression profiles in human brain organoids and those of other primates identified the mTOR pathway, which is specifically activated in human radial glial cells, as a signaling pathway that is unique to humans (Pollen et al., 2019). Furthermore, in the field of disease modeling, this technology has been used to demonstrate that impairment of N-cadherin/β-catenin signaling is a crucial mechanism underlying lissencephaly caused by deficiency of the LIS1/NDEL1/14.3.3ϵ complex (Iefremova et al., 2017). Investigation of the mechanism of Zika virus infection platforming organoids identified neural progenitor cells as the cell population that is primarily affected by infection (Qian et al., 2016). Additionally, brain organoid-based modeling of fetal brain hypoxia has revealed that intermediate progenitors in the subventricular zone are mainly damaged under hypoxic conditions, which was confirmed in patient samples (Paşca et al., 2019).

Thus, the multicellular and layered human brain-like structure of brain organoids has greatly contributed to the understanding of the mechanisms underlying human brain development and diseases.

## Current Analysis of Human Neural Circuits in Brain Organoids

Organoid technology has only been applied in a few studies on the process of human neural circuit formation. To establish functional neural circuits, neurons precisely follow stepwise processes, including cell migration, axonal projection, dendritic growth, synapse formation and synapse elimination. Among those steps, so far, several organoid-based researches on cell migration, axonal projection and synapse formation have been published (Birey et al., 2017; Xiang et al., 2019). In this section, we discuss the use of human brain organoids for the analysis of neural circuits.

In humans as well as rodents, newborn neurons migrate radially or tangentially to a preprogrammed destination in the cerebral cortex and form a specific neural network (Marín et al., 2010; Bartolini et al., 2013; Paredes et al., 2016). In humans, excitatory glutamatergic neurons differentiate from radial glial cells around the lateral ventricles at approximately gestational week 15 and undergo directional radial migration to establish a six-layered structure (Das and Takahashi, 2018; Larkum et al., 2018). Electrophysiological analysis conducted in the mouse brain revealed that pyramidal neurons in layers II, III, IV, and V form local excitatory microcircuits between cortical layers, with prominent excitatory pathways from layer II/III to layer V (Thomson and Bannister, 2003; Hooks et al., 2011). However, these local excitatory circuits have not been analyzed in detail in human samples. Human brain organoids are able to partially recapitulate radial migration, producing cerebral cortex-like neuronal layers. Mice and humans share the same basic layered cortical structure composed of distinct neurons expressing markers specific for each of the six cortical layers: REELIN for layer I, CUX1 for layer II, BRN2 for layer III, SATB2 for layer IV, CTIP2 for layer V and TBR1 for layer VI (Leone et al., 2008; Qian et al., 2016). Forebrain organoids, which can be generated by the fine tuning of growth factors, reportedly include neurons expressing marker genes for each of the six neuronal layers, although the boundaries of cortical neural layers in forebrain organoids are not as distinct as those in the human fetal brain (Qian et al., 2016). The expression of layer-specific marker genes indicates that neurons in forebrain organoids recapitulate the gene expression profile of the human brain. Satb2, Ctip2, and Tbr1 are known to be transcription factors that determine the identity of neurons and affect the pattern of axonal projection and connectivity in rodents (Leone et al., 2008). Thus, for example, Satb2-expressing neurons in forebrain organoids likely establish, perhaps only partially, intracortical circuits that mimic those in the human cerebral cortex.

**FIGURE 1:**
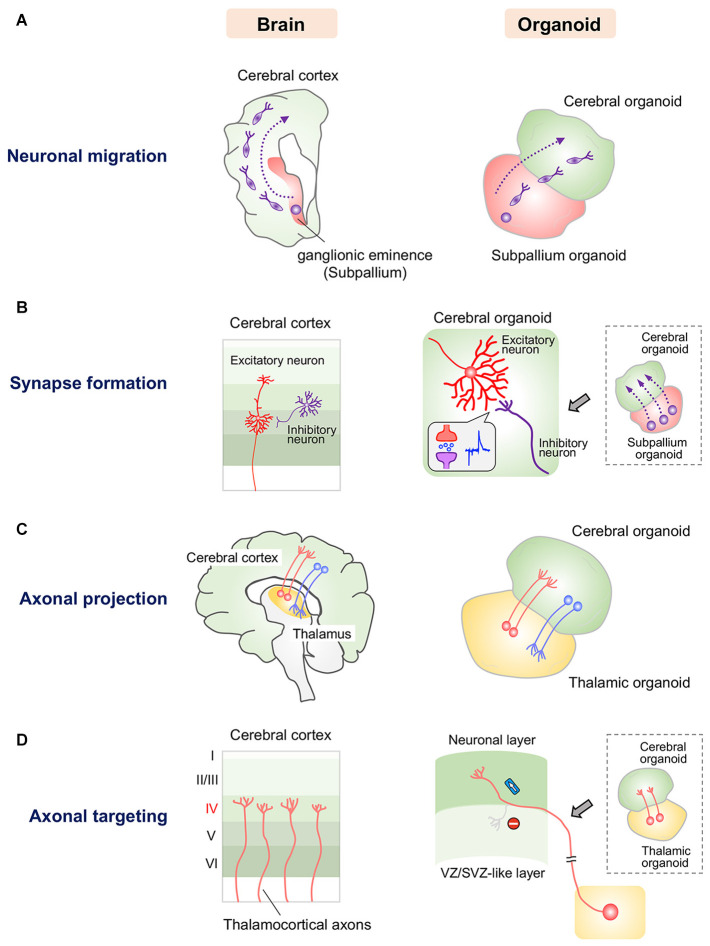
Current approaches for modeling human neuronal circuit formation with fused brain organoids. **(A)** Left: Tangential migration of GABAergic interneurons from the ganglionic eminence to the cerebral cortex in the human brain. Right: GABAergic interneurons in subpallium organoids migrate into the cerebral organoid in fused organoids. **(B)** Left: Synapse formation between excitatory and inhibitory neurons in the human cerebral cortex. Right: GABAergic interneurons that migrate from subpallium organoids form functional synapses with excitatory neurons in cerebral organoids. **(C)** Left: Reciprocal axonal projection between the thalamus and cerebral cortex in the human brain. Right: Thalamic and cortical neurons reciprocally project axons into the other organoid in the fused system. **(D)** Left: Thalamocortical axons specifically innervate layer 4 of the somatosensory cortex. Right: Axons of thalamic organoids selectively innervate the neuronal layer but not the VZ/SVZ-like layer, in which neural stem/progenitor cells reside, in the fused cerebral organoid system.

In humans, at approximately gestational week 17, inhibitory GABAergic interneurons originating from the ganglionic eminence migrate tangentially into the cerebral cortex (Figure 1A), connect to glutamatergic excitatory neurons via synapses, and form microcircuits in the cerebral cortex (Rakic and Zecevic, 2003). Mimicking tangential migration in organoids is more difficult than mimicking radial migration because tangentially migrating neurons travel a long distance between distinct components. In the fetal brain, the ganglionic eminence and the cerebral cortex develop in a highly synchronized manner that cannot be completely recapitulated simultaneously by current organoid technology. Thus, fused organoids composed of the glutamatergic excitatory neuron-rich cerebral organoids and GABAergic interneuron-rich subpallium organoids induced by fine tuning of growth factors have been generated to model neuronal migration between distant components (Birey et al., 2017; Xiang et al., 2017; Figure 1A). GABAergic interneurons reportedly migrate from subpallium organoids to cerebral organoids and form synapses with local glutamatergic neurons (Birey et al., 2017; Xiang et al., 2017). This observation indicates the existence of a mechanism allowing orientation of neuronal migration followed by synapse formation in the fused organoid system. Moreover, in these fused organoids, stimulation of migrated GABAergic interneurons induced evoked inhibitory postsynaptic currents in glutamatergic neurons, while stimulation of glutamatergic neurons induced evoked excitatory postsynaptic currents in GABAergic interneurons (Birey et al., 2017; Figure 1B). These findings indicate that migrated GABAergic neurons are functionally integrated into local excitatory circuits in cerebral organoids (Birey et al., 2017). Additionally, human cerebral organoids and thalamic organoids have been combined to model the thalamocortical circuit (Xiang et al., 2019; Figure 1C). Cortical neurons and thalamic neurons extend axons and form synapses with each other, recapitulating the reciprocal thalamocortical axonal projections involved in the transmission of sensory and motor information in the human brain (López-Bendito and Molnár, 2003; Xiang et al., 2019). Interestingly, thalamic organoid-derived axons selectively innervate the upper neuronal layers of cortical organoids, avoiding the lower VZ/SVZ-like region in which NSCs reside (Figure 1D). This selectivity indicates that like the human brain, fused thalamocortical organoids possess orchestrated molecular machinery for proper axonal guidance and targeting.

The above mentioned six-layered forebrain organoids may be fused with other region-specific organoids in the future for analysis of the detailed patterns of neuronal migration, axonal innervation and synapse formation (Qian et al., 2016; Birey et al., 2017; Xiang et al., 2019). In addition, we may be able to utilize genome editing technology to analyze the fine structures of human neural circuits in brain organoids by labeling specific types of neurons with fluorescent reporter genes. We are also able to investigate the molecular mechanisms underlying human neural circuit formation through genome editing-mediated knockout of candidate genes. Moreover, the newly developed microelectrode array system will help us measure neural activity at the circuit level in organoids and facilitate functional mapping of neural networks formed in organoids.

## Current Obstacles and Perspectives for Organoid-Based Analysis of Neural Circuits

**FIGURE 2:**
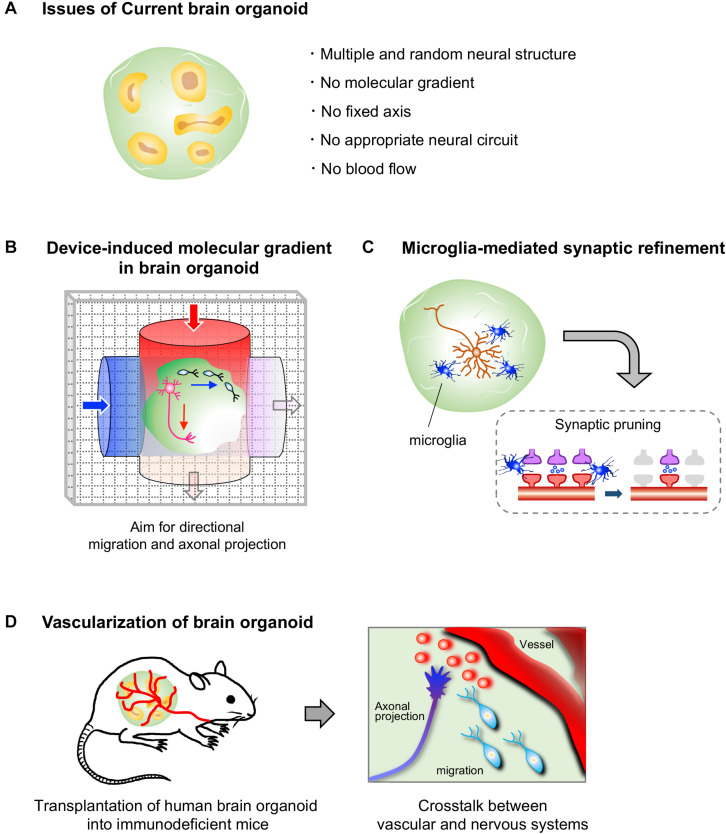
Current limitations to brain organoids and prospective approaches. **(A)** Human brain organoids generated by current protocols lack a self-generating molecular gradient, as well as blood flow. These limitations hamper the formation of a structural axis assembled by orderly-positioned brain components; therefore the formation of appropriate neural circuits in brain organoids is impeded. **(B)** A device that generates molecular gradients would be effective in inducing directional migration and axonal projections, as well as a structural axis. Embedding of cells that secrete a certain molecule such as morphogens may also help to generate molecular gradients in brain organoids (not depicted). **(C)** Human brain organoids that contain microglial cells may allow us to analyze a process of synaptic refinement, such as microglia-mediated synapse pruning, in human neural circuits. **(D)** The transplantation of human brain organoids into highly vascularized organs of immunodeficient mice may allow us to analyze vascular system-mediated neural circuit formation in humans.

As described above, there have been some reports on neuronal migration, axonal projection and synapse formation in fused human brain organoids, although the molecular mechanisms underlying these events have not been studied (Birey et al., 2017; Xiang et al., 2019). During neural development, spatiotemporally coordinated expression of various guidance molecules and cell adhesion molecules is essential for establishing precise neural circuits. These molecules are expressed by specific cells and sometimes form local gradients in restricted brain regions, enabling proper neuronal migration, axonal guidance, and synapse formation. However, the organoids generated by the currently available protocols possess multiple randomly positioned neural tube-like structures and therefore lack a fixed structural axis (Figure 2A). Morphogen gradients, such as sonic hedgehog (SHH) gradients and fibroblast growth factor gradients (Stevens et al., 2010; Oosterveen et al., 2012; Bökel and Brand, 2013), in organoids are indispensable for establishing appropriately positioned brain components that lead to precise circuit formation, especially in the early stage of neural differentiation. Nevertheless, the generation of morphogen gradients in current brain organoid protocols has rarely been successful.

To generate brain organoids bearing molecular gradients, we should first focus on relatively simple neural structures, such as the spinal cord. In the developing spinal cord, the gradients of morphogens, such as SHH and bone morphogenetic protein (BMP), play key roles in the determination of regional identity in the early developmental stage (Shirasaki and Pfaff, 2002; Ulloa and Briscoe, 2007; Osseward and Pfaff, 2019). The balance between roof plate-derived BMP and floor plate-derived SHH defines regional identity along the dorsoventral axis in the spinal cord. Subsequently, each neural progenitor cell along the dorsoventral axis acquires a region-specific expression profile of transcription factors that leads to differentiation of the cell into a specific neuronal subtype (Shirasaki and Pfaff, 2002; Ulloa and Briscoe, 2007; Osseward and Pfaff, 2019). Then, each neuron expresses a set of cell type-specific receptors for chemoattractants and cell adhesion molecules to form each circuit.

Although several groups have reported the generation of spinal cord organoids, these spinal cord organoids lack the proper molecular gradients (Ogura et al., 2018; Duval et al., 2019). Thus far, organoids mimicking the dorsal and ventral parts of the spinal cord cannot be induced simultaneously; therefore, these organoids fail to recapitulate the exact regionality and cell type specificity of the spinal cord. Because of these limitations, analysis of neural circuits seems impossible in these organoids (Ogura et al., 2018; Duval et al., 2019). The generation of spinal cord organoids with morphogen gradients may lead to region-specific expression of transcription factors and subsequent autonomous differentiation into specific neuronal subtypes. In addition, morphogen gradients may also produce surrounding cells such as floor plate cells, which secrete several axonal guidance molecules, including netrin and slit.

Newly developed devices, such as microfluidic devices and molds, are attracting interest for studying morphogen gradient axes in organoids (Figure 2B). A group reported successful modeling of the structure of the neural tube by the combined use of a mold, which enables the formation of a tube-like structure by pluripotent stem cells, and a microfluidic device, which produces a Wnt gradient that is critical for rostrocaudal axis formation in the neural tube (Rifes et al., 2020). In this study, a tube-like structure acquired region-specific gene expression profiles along the rostrocaudal axis under the action of a Wnt gradient. The rostral region exposed to low concentration of Wnt showed the marked expression of OTX2, a highly expressed gene in the developing forebrain and midbrain, while the caudal region exposed to high concentration of Wnt expressed GBX2, a highly expressed gene in the developing hindbrain. Additionally, transplantation of morphogen-secreting cells into the organoid may allow the formation of the dorsoventral axis. Indeed, embedding of SHH-secreting human pluripotent stem cells at one pole of a cerebral organoid was shown to successfully induce an SHH gradient and subsequent gene expression topography in one organoid, even though multiple neural structures randomly emerged in the organoid (Cederquist et al., 2019). Moreover, SHH-secreting floor plate-like tissue can be induced from pluripotent stem cells (Fasano et al., 2010).

Thus, by utilizing existing technologies, it may be feasible to generate spinal cord organoids with rostrocaudal and dorsoventral axes in the near future. If we obtain such organoids, we may recapitulate complicated spinal neural circuits, such as a circuit of commissural neurons that project axons that first extend ventrally, cross the midline, and subsequently turn orthogonally toward the anterior direction to form synapses with targets.

## Potential Modeling of the Synaptic Pruning With Brain Organoids

In the course of neural circuit formation, neurons transiently form excess synapses. In rodents, some inactive synapses are removed after birth, mainly by microglial cells, which selectively leave functional synapses (Paolicelli et al., 2011; Schafer et al., 2012). Brain organoids have not yet been utilized to investigate the pruning processes in humans. To model this phenomenon with brain organoids, both microglial cells and firing neurons with synapses must be included in a single organoid because microglial cells engulf the synapses of neurons with low neuronal activity (Schafer et al., 2012; Figure 2C). Neuronal firing has already been observed in some brain organoids (Giandomenico et al., 2019; Trujillo et al., 2019; Eura et al., 2020), and synapse formation between neurons has also been observed in long-cultivated organoids and fused organoids (Birey et al., 2017; Trujillo et al., 2019; Xiang et al., 2019). Microglial cells are not expected to be present in brain organoids because microglial cells are yolk sac-derived mesodermal cells (Gomez Perdiguero et al., 2015), whereas brain organoids are established by neuroectodermal induction. However, microglial cells expressing IBA-1, a microglial marker, have been detected in the brain organoids induced by the autonomous differentiation protocol without fine tuning of growth factors (Lancaster et al., 2013; Ormel et al., 2018). It is possible that, in this protocol, a few pluripotent cells differentiate into mesodermal linage cells as a result of weak neuroectodermal induction without fine tuning. Additionally, methods for inducing pure microglial populations from ES/iPSCs are already available (Muffat et al., 2016). Thus, theoretically, we are now able to model the human brain with microglia through appropriate selection of a brain organoid protocol or by mixing human brain organoids with microglial cells induced from pluripotent stem cells. Accordingly, human brain organoids are expected to become a powerful tool for analyzing the molecular mechanism underlying microglia-mediated synaptic pruning. We can also manipulate neuronal firing in organoids through optogenetic tools and knockout specific genes by genome editing, which will further facilitate research on synaptic pruning. The establishment of a method for inducing organoids along with abundant microglial cells will also facilitate the development of more sophisticated brain organoids because microglial cells have been shown to support the proliferation of NSCs and neuronal survival in rodents (Ueno et al., 2013; Matsui and Mori, 2018).

## Potential Analysis of Vascular System-Mediated Neural Circuit Formation With Brain Organoids

Blood vessels are another major non-neuronal component of the brain and are mainly composed of endothelial cells, smooth muscle cells and pericytes. In perinatal mice, neuroblasts around the corpus callosum require blood vessels as scaffolds for radial migration to cortical neuronal layers (Le Magueresse et al., 2012). Blood vessels also serve as scaffolds for elongating neurites in the cortical germinal zone (Stubbs et al., 2009). Several vascular-derived molecules have been identified to regulate neural circuit development. GABA and VEGF-A secreted by vascular vessels control tangential migration of interneurons from the medial ganglionic eminence to the cerebral cortex in mice (Inada et al., 2011; Won et al., 2013; Barber et al., 2018). Endothelin derived from endothelial cells directs the extension of sympathetic axons and their innervation of the external carotid artery (Makita et al., 2008). In addition to playing a regulatory role in migration, VEGF also facilitates synaptogenesis in the cerebral cortex (Wu et al., 2019). However, blood vessel-mediated neural circuit formation has yet to be confirmed in humans.

While several vascular cells, such as endothelial cells and smooth muscle cells, can be observed in brain organoids (Logan et al., 2020), a self-organizing vessel structure is absent in brain organoids generated with current protocols. On the other hand, several groups have achieved vascularization of human brain organoids by utilizing the mouse vascular system via transplantation of human brain organoids into the brains or limbs of immunodeficient mice (Daviaud et al., 2018; Mansour et al., 2018; Cakir et al., 2019; Shi et al., 2020). Thus, these vascularized organoids are expected to contribute to analyze the effect of vascularization on neural circuit formation in humans in the near future. Brain organoid-based modeling may provide an opportunity for studying the interaction between human vascular and neuronal systems, as no methods are currently available (Figure 2D).

## Concluding Remarks

Research on human neural circuits in organoids has just begun. Fused organoids partially model some steps in the process of neural circuit formation. However, there are still many obstacles to mimicking human neural circuits in brain organoids. Thus, we need more sophisticated brain organoids containing a combination of functional neuronal types, brain residential non-neuronal cells, vascular structures, and perhaps most importantly, regionalizing mechanisms. Once we overcome these obstacles, we will be able to elucidate the human-specific molecular mechanisms that underlie neural circuits, leading to future modulation of human neural circuits.

## Author Contributions

TM and KK contributed to the writing and editing of the manuscript. YT contributed to the editing of the manuscript. All authors contributed to the article and approved the submitted version.

## Conflict of Interest

The authors declare that the research was conducted in the absence of any commercial or financial relationships that could be construed as a potential conflict of interest.
